# The role of Chinese medical teams in bridging healthcare gaps in Africa: a scoping review

**DOI:** 10.1186/s41256-025-00420-2

**Published:** 2025-06-16

**Authors:** Emmanuel Kwasi Afriyie, Samuel Egyakwa Ankomah, Duqiao Li, Yuqing Guo, Huijuan Liang, Dadong Wu, Dong Xu

**Affiliations:** 1 Acacia Lab for Implementation Science, School of Health Management, Southern Medical University, Guangzhou, China; 2 Komfo Anokye Teaching Hospital, Kumasi, Ashanti Region Ghana; 3 Department of Management, University of Cape Coast, Cape Coast, Ghana; 4 Zhujiang Hospital (School of Second Clinical Medicine College), Southern Medical University, Guangzhou, China; 5 School of Healthcare Management, Inner Mongolia Medical University, Hohhot, China; 6 Shenzhen Maternity and Child Healthcare Hospital, Southern Medical University, Shenzhen, China; 7 Acacia Lab for Implementation Science, SMU Institute for Global Health (SIGHT) and Center for World Health Organisation Studies, School of Health Management and Dermatology Hospital of Southern Medical University, Guangzhou, China

**Keywords:** Chinese medical team, Africa, Healthcare gaps, Modality, Effectiveness, Scoping review

## Abstract

**Background:**

Sub-Saharan Africa has faced profound healthcare challenges, including severe shortages of professionals and infrastructural deficits. Despite significant international aid, the full impact of Chinese Medical Teams (CMTs) in addressing these issues had remained underexplored. This scoping review aimed to synthesise existing literature on the role of CMTs in Africa, identifying key drivers, barriers, and gaps in research that could enhance the effectiveness of these programmes.

**Methods:**

A comprehensive search was conducted across major English and Chinese databases up to February 2023, following the Arksey and O'Malley framework and adhering to the PRISMA-ScR checklist. Studies providing qualitative or quantitative insights into the modality, effectiveness, and challenges of CMTs were included. Thematic analysis, supported by NVivo 11 software, was used to synthesise the findings.

**Results:**

The review included 20 English articles and 27 Chinese articles from 2009 to 2022, highlighting CMTs’ significant role in improving healthcare through direct medical assistance, training of local healthcare workers, and infrastructure development. Key drivers of CMT initiatives included diplomatic goals, economic cooperation, and humanitarian efforts. Conversely, operational challenges such as cultural differences, language barriers, and infrastructural inadequacies were prominent.

**Conclusions:**

CMTs have effectively addressed healthcare disparities in Africa through a distinctive, government-led, and non-conditional programme. Their flexible, long-term engagement has strengthened healthcare systems across underserved regions, offering a model for sustainable global health aid. However, challenges such as cultural barriers and logistical constraints suggest a need for improved cultural competency and flexible staffing. Further empirical research, particularly involving African researchers, is essential to fully understand CMTs' long-term impact and refine strategies for future international health initiatives that align with local needs.

**Supplementary Information:**

The online version contains supplementary material available at 10.1186/s41256-025-00420-2.

## Introduction

Access to healthcare is a fundamental human right [1], yet ensuring this right remains a significant challenge in many sub-Saharan African countries [2–4]. Despite the ambitious target set by Sustainable Development Goal 3 to achieve universal health coverage by 2030, this objective continues to be elusive for sub-Saharan Africa [5], which accounts for nearly 24% of the global burden of disease [6]. Economic constraints are a major contributor to the challenge, as approximately 40% of the sub-Saharan African population still lives in extreme poverty (surviving on less than US$1.9 per day) [7]. Weak healthcare infrastructure exacerbates this situation, characterised by shortages of healthcare facilities, workers and medical equipment [8]. The World Health Organisation (WHO) reports that 36 out of the 47 countries in the African region face shortages of doctors, nurses and midwives, representing the highest rate of healthcare professional shortages among all WHO regions [9].

Despite multilateral and bilateral aid from various countries, substantial gaps in healthcare infrastructure and services persist in Africa [10]. As one of the key donors, China has been addressing these gaps by providing medical aid to Africa, especially through the deployment of medical teams [11]. These teams deliver on-the-ground medical support, share expertise, and train local healthcare workers [12]. From 1963 to 2019, Chinese medical teams (CMTs) have served more than 180 million patients across 51 African countries, offering over 400 million outpatient consultations, 20 million inpatient treatments, and 1.6 million surgeries [13, 14]. In total, over 20,000 medical professionals have been dispatched to African countries [14]. During the outbreaks of Ebola virus disease between April and October 2014, the substantial emergency aid provided by CMTs to West African countries totalled 750 million Chinese yuan (approximately US$ 123 million) [15]. Additionally, from 2020 to 2021, CMTs significantly contributed to combating COVID-19 in African countries through the provision of vaccines, medical supplies, and personal protective equipment [12].

Despite the long and extensive involvement of CMTs in Africa, a comprehensive understanding of their efforts in bridging local healthcare gaps remains unclear. This gap in knowledge may hinder the optimisation of CMT programmes, thereby limit their overall effectiveness. Herein, this scoping review aims to consolidate existing literature on the impact of CMTs in mitigating healthcare access disparities in African countries. Specifically, it examines (1) the modality and effectiveness of CMT programmes in Africa; (2) the primary drivers and barriers influencing CMTs in their efforts to bridge local healthcare gaps. The anticipated findings are expected to provide valuable insights into the roles and effectiveness of CMTs, thereby supporting strategic planning and policy-making regarding China’s foreign medical aid. Additionally, the review aims to pinpoint any research gaps that require further exploration.

## Methods

This study employed the scoping review methodology outlined by Arksey and O'Malley [16] to synthesise both qualitative and quantitative evidence on the activities of CMTs in African countries. A detailed protocol was developed (Supplementary Material: Appendix 1), and the reporting adhered to the Preferred Reporting Items for Systematic Reviews and Meta-Analyses extension for scoping reviews (PRISMA-ScR) checklist [17] to ensure transparency in methodology, analytical processes, and findings (Supplementary Material: Appendix 2).

### Search strategy

A comprehensive search was conducted in major English and Chinese peer-reviewed databases, including PubMed, Web of Science, ScienceDirect, Scopus, Cumulative Index to Nursing and Allied Health Literature, China National Knowledge Infrastructure and Wanfang Data, in February 2023 to identify relevant publications from inception until the search date. To ensure inclusivity, broad search terms encompassing various aspects of CMT activities were used, allowing key themes to emerge naturally rather than being restricted by predefined outcomes. For searches in English databases, the free-text terms were applied: (("Medical assistance"OR"medical team"OR"medical program"OR"bilateral assistance"OR"bilateral support"OR"bilateral funding"OR"bilateral aid"OR"CMT"OR"CMA"OR"foreign aid"OR"foreign assistance"OR"medical mission"OR"medical group"OR"medical intervention"OR"medical service") AND (China OR Chinese)) AND (Africa OR"Low-and middle-income countries"OR"LMICs"). The search string was first tested in PubMed and subsequently adapted to other databases. The full search strategies for all databases are detailed in Supplementary Material: Appendix 3. Additionally, the reference lists of included publications were screened for relevant articles not otherwise captured.

### Inclusion and exclusion criteria

This review included English and Chinese publications that provided qualitative or quantitative evidence on the impact of CMTs in Africa. Articles were eligible if they discussed the modality and effectiveness of CMT programmes or explored the facilitators and barriers these programmes encounter in addressing local healthcare gaps. Articles that were not directly related to medical aid, general discussions on China’s medical assistance to Africa without specific reference to CMTs, conference proceedings, non-substantive commentaries or opinion pieces lacking analytical depth, abstracts, book reviews, and publications for which full texts were inaccessible were excluded.

### Study selection

We imported all retrieved records into Endnote X9 software (Clarivate, Philadelphia, U.S.) and removed duplicates. The inclusion and exclusion criteria were pilot-tested on 25% of the articles and refined for clarity and applicability. EKA and SEA independently screened the titles and abstracts of the English publications and then compared their results. DL and YG performed similar tasks for the Chinese publications. Both screening groups regularly consulted with DW, an experienced researcher in scoping review, to resolve discrepancies, ensuring that consensus was reached before continuing. This approach, which involved multiple screeners, was pivotal in reducing biases and errors [18].

### Quality assessment of included publications

The quality of the included articles was appraised using the Joanna Briggs Institute Critical Appraisal Checklist [19]. Each criterion on the checklist was evaluated for every study, assigning ratings of"Yes","No","Unclear", or"Not applicable". Articles without any"No"or"Unclear"ratings were considered"Strong"in quality. Those with one to three such ratings were categorised as"Moderately strong", while those with more than three were deemed"Weak". Only articles rated as"Strong"or"Moderately strong"were included for analysis. Detailed appraisals of each publication are available in Supplementary Material: Appendix 4.

### Data extraction

A purposefully designed Microsoft Excel spreadsheet was used to extract data from included articles, including title, year of publication, first or corresponding author’s country, journal impact factor, article type and study design, data source, targeted country, targeted issue, and CMT intervention. EKA, SEA, DL, and YG first tested the data extraction form on 3 English/Chinese articles individually and consulted with DW to resolve discrepancies. Then, EKA and SEA extracted data from English articles, and DL and YG charted Chinese articles.

### Evidence synthesis and analysis

Thematic analysis was employed to examine the data [20], using NVivo 11 qualitative data analysis software (QSR International, Burlington, U.S.) to organise the included articles [21]. Data analysis was guided by the study objectives, with themes emerging inductively from the literature rather than being predefined. To ensure the trustworthiness and validity of the analytical processes, a debriefing technique was used, which involved regular discussions among the research team to resolve discrepancies, refine coding structures, and ensure consistency in theme identification [22]. EKA and SEA independently coded the included English articles, while DL and YG coded the Chinese articles. Throughout the process, findings were regularly discussed with DW and DX to refine interpretations and address any inconsistencies. The final themes identified were: (a) Modality: programme location, programme duration, collaborative initiatives, resources and expertise; (b) Effectiveness: meeting local healthcare needs, improving the quality of care, capacity building; (c) Major drivers: political and diplomatic goals, economic cooperation, humanitarian aid; (d) Barriers: infrastructure and resource disparities, cultural differences, language barriers.

## Results

### Selection of sources of evidence

**Fig. 1:**
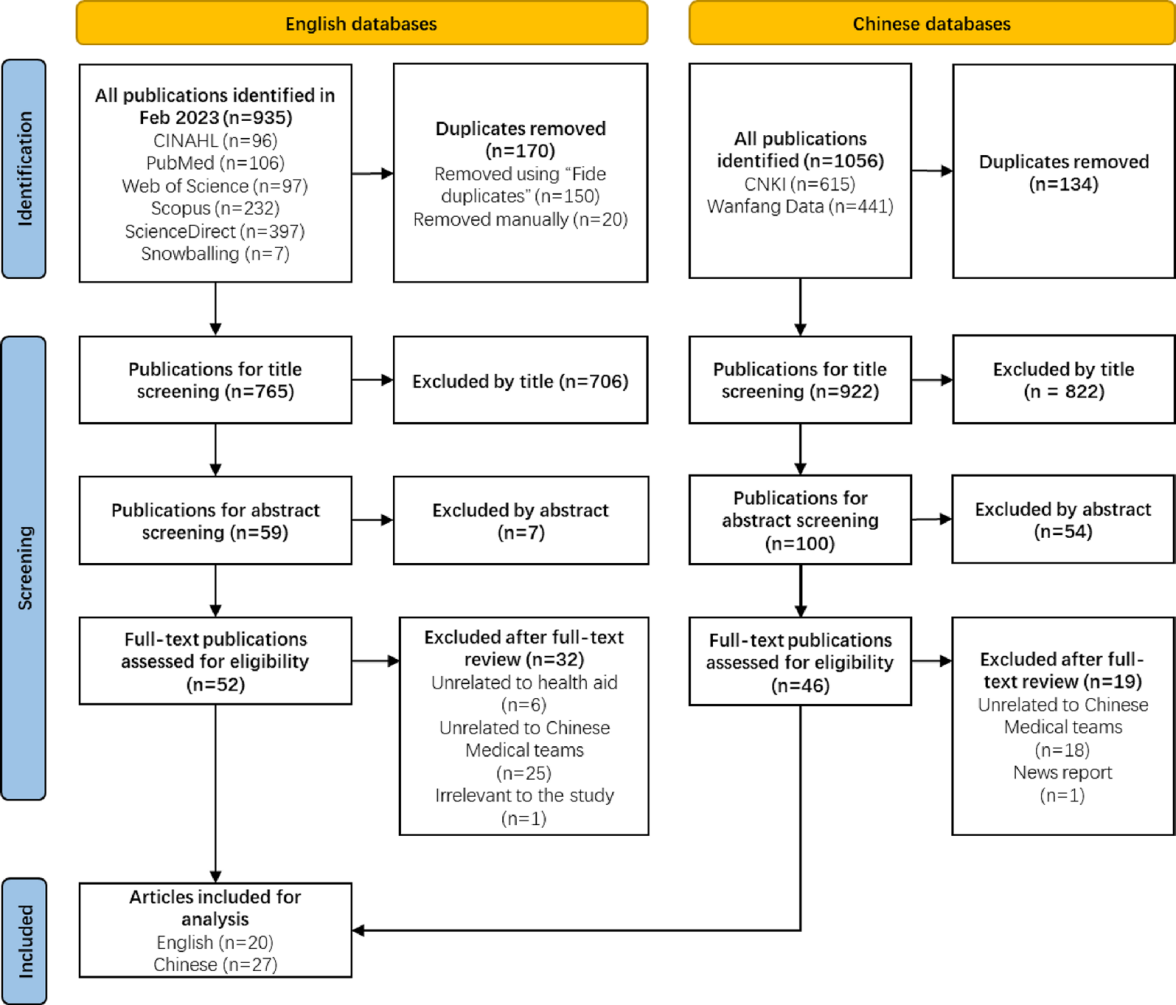
PRISMA-ScR flow diagram of the study selection process

The study selection process is illustrated in the PRISMA-ScR flow diagram in Fig. 1. Initially, a total of 935 English and 1056 Chinese publications were identified. After duplicates were removed, the numbers decreased to 765 and 922, respectively. Subsequent title and abstract screening further narrowed the selection to 52 English and 46 Chinese articles for full-text review. Finally, 32 English articles (6 unrelated to medical aid, 25 unrelated to CMTs, and 1 unrelated to the study) and 19 Chinese articles (18 unrelated to CMTs and 1 news report) were excluded, leaving 20 English and 27 Chinese articles for the final synthesis.

### Characteristics of studies included

Table 1 outlines the characteristics of the 20 English articles included, published between 2009 and 2022. Most of these articles (n = 15) explored the impact of CMTs across multiple African countries. Three articles specifically focused on individual countries. Two articles conducted cross-country comparative analyses. Notably, researchers from China and/or high-income countries (HICs) (U.S., Canada, Sweden) were the first or corresponding authors in 18 of the 20 articles. In comparison, only 2 articles were published collaboratively by researchers from China and other LMICs (Bangladesh and Cameroon). More than half of the articles (n = 11) were published in journals without an impact factor at the time of publication, whereas only 1 review appeared in The Lancet in 2014 (Impact factor: 44.002).

**Table 1:** Characteristics of the 20 English articles included for evidence synthesis and analysis

Title	Year of publication	First or corresponding author’s country	Journal (IF in the year of publication)	Article type and study design	Data source	Targeted country	Targeted issue	CMT intervention
China-Africa Cooperation Initiatives in Malaria Control and Elimination [28]	2014	China	Advances in Parasitology (IF: 4.829)	Review article	Literature review	Multiple countries	Infrastructure development Malaria control	Establishment of malaria control centres Supply of medicines and equipment Medical team assistance
China's foreign aid for global poverty alleviation: artemisinin-based combination therapies against malaria in Togo [38]	2021	China	Global Health Journal (IF: 0)	Review article	The malaria profile in Togo; literature review	Togo	Malaria control	Training of local healthcare professionals Building of malaria cooperative laboratory Provision of anti-malaria drugs
China's engagement with development assistance for health in Africa [33]	2017	Bangladesh & China	Global Health Research and Policy (IF: 0)	Original research: quantitative	Features of 531 health-related projects undertaken between 2000 and 2013	Multiple countries	Infrastructure development Malaria control Tuberculosis control Highly infectious diseases such as Influenza, Lasa Fever, sexual and Reproductive Health	Financial aid Supply of medicines and equipment Training of local healthcare professionals Medical team assistance Health education and promotion
Challenges and Opportunities in China's Health Aid to Africa: Findings from Qualitative Interviews in Tanzania and Malawi [32]	2020	U.S	Globalization and Health (IF: 2.525)	Original research: Qualitative	Interviews with 29 key stakeholders in Malawi (20 Malawian and 9 Chinese) and 29 key stakeholders in Tanzania (13 Tanzanians and 16 Chinese)	Malawi and Tanzania	Infrastructure development Health workforce	Medical team assistance Training of local healthcare professionals
Influencing Factors for Expatriation Willingness of Chinese Medical Aid Team Members in Africa: A Qualitative Descriptive Study [30]	2020	China	International Journal of Environmental Research and Public Health (IF: 2.849)	Original research: Qualitative	Interviews with hospital directors, local Health and Family Planning Commission (HFPC) officers, and Chinese medical aid team members	Multiple countries	Highly infectious diseases such as HIV and dengue fever Ebola outbreak Malaria control	Medical team assistance
China's distinctive engagement in global health [24]	2014	China	The Lancet (IF: 44.002)	Review article	Literature review; interviews with former officials, CMT members, and key provincial authorities	Multiple countries	Infrastructure development Malaria control	Financial aid Supply of medicines and equipment Medical team assistance Training of local healthcare professionals
Enhancing China-Africa Health Cooperation for a Healthier and Safer World: A Multilateral Perspective [12]	2022	China	China Quarterly of International Strategic Studies (IF: 0)	Original research: qualitative	Structured literature review; semi-structured questionnaires and interviews with officials from African countries, multilateral organisations, as well as Chinese policymakers and researchers	Multiple countries	HIV/AIDS Tuberculosis control Ebola and COVID-19 outbreaks	Medical team assistance Health education and promotion Immunisation Provision of vaccines and personal protective equipment
Chinese medical teams in Africa: a flagship program facing formidable challenges [14]	2019	China & U.S	Journal of Global Health (IF: 3.079)	Viewpoint	Literature review; meetings/workshops on China’s health aid programmes; 17 interviews with international donors, Ministry of Health officials, and CMT members	Tanzania and Ghana	Infrastructure development Health workforce	Medical team assistance Building medical facilities Supply of medicines and equipment
China's engagement in global health governance: A critical analysis of China's assistance to the health sector of Africa [29]	2014	China	Journal of Global Health (IF: 3.559)	Viewpoint	Literature review; books; government official documents and data; working papers from non-state organisations and think tanks	Multiple countries	Infrastructure development Healthcare services	Medical team assistance Financial aid
China's provincial diplomacy to Africa: Applications to health cooperation [27]	2014	U.S	Contemporary Politics (IF: 0)	Review article	Literature review	Multiple countries	HIV/AIDS Tuberculosis control Malaria control Infrastructure development Health workforce Healthcare services	Financial aid Supply of medicines and equipment Medical team assistance Training of local healthcare professionals
China's African policy in the post-Cold War era [11]	2009	China	Journal of Contemporary Asia (IF: 0)	Review article	Policy documents; Literature review	Multiple countries	N/A	N/A
Domestic politics and China's health aid to Africa [25]	2014	China	China: An International Journal (IF: 0)	Review article	Literature review	Multiple countries	Ebola outbreak Health workforce Infrastructure development	Medical team assistance Financial aid Supply of medicines and equipment Training of local healthcare professionals
China's health assistance to Africa: opportunism or altruism? [15]	2016	U.S	Globalization and Health (IF: 2.54)	Review article	Literature review	Multiple countries	Health workforce Healthcare services	Medical team assistance Supply of medicines and equipment Training of local healthcare professionals
The value of China-Africa health development initiatives in strengthening the “One Health” strategy [31]	2017	Cameroon & China	Global Health Journal (IF: 0)	Review article	Literature review	Multiple countries	Malaria control Highly Infectious diseases such as measles, filariasis, and schistosomiasis SARS and Ebola outbreaks	Medical team assistance Building medical facilities Supply of medicines and equipment Health education and promotion
China's Aid Policies in Africa: Opportunities and Challenges [34]	2010	Canada	The Round Table: The Commonwealth Journal of International Affairs (IF: 0)	Review article	Literature review	Multiple countries	N/A	N/A
Old bottle new wine? The evolution of China’s aid in Africa 1956–2014 [26]	2019	China	Third World Quarterly (IF: 0)	Review article	Features of China’s aid projects in Africa from 1956 to 1999	Multiple countries	Infrastructure development	Financial aid Supply of medicines and equipment Medical team assistance
Chinese military medical teams in the Ebola outbreak of Sierra Leone [35]	2016	China	Journal of the Royal Army Medical Corps (IF: 0.662)	Original article: Descriptive study	Features of the Chinese Military Medical Teams (CMMTs) rescue mission in Sierra Leone 2014–2015	Sierra Leone	Ebola outbreak Infrastructure development	Medical team assistance Building Ebola cases holding area Training of local healthcare professionals
China’s role as a global health donor in Africa: what can we learn from studying under-reported resource flows? [23]	2014	U.S., China	Globalization and Health (IF: 2.54)	Original research: Quantitative	Features of 255 health, population, water, and sanitation (HPWS) projects undertaken from 2000–2012	Multiple countries	Infrastructure development Malaria control	N/A
Experiences and challenges in the health protection of medical teams in the Chinese Ebola Treatment Centre, Liberia: A Qualitative study [36]	2018	China	Infectious Diseases of Poverty (IF: 0)	Original research: Qualitative	Interviews with 15 informants from the People’s Liberation Army of China medical team which operated the Chinese Ebola Treatment Centre from 2014 to 2015	Liberia	Ebola outbreak	Medical team assistance Training of local healthcare professionals
Determinants of China’s development assistance for Health at the sub-national level of African countries (2006–2015) [37]	2018	Sweden & China	Infectious Diseases of Poverty (IF: 0)	Original research: Quantitative	Features of China's Development Assistance for Health (DAH) Project 2006–2015	Multiple countries	Infrastructure development Malaria control	Medical team assistance Building hospitals and Anti-malaria centres Training of local healthcare professionals Supply of medicines and equipment

*IF* impact factor, *CMT* Chinese medical team

The included English articles comprised 10 reviews, 2 viewpoints, and 8 original articles, of which 5 were qualitative and 3 were quantitative. These studies spanned a broad spectrum of health-related issues in African countries, demonstrating the multifaceted contributions of CMTs across the continent. Topics included essential healthcare infrastructure development (n = 12), malaria control (n = 9), the Ebola outbreak (n = 6), health workforce (n = 5), highly infectious diseases including HIV (n = 5), tuberculosis control (n = 3), healthcare service enhancement (n = 3), the COVID-19 pandemic (n = 1), and SARS (n = 1). The interventions undertaken by CMTs, as reported in the articles, included medical team assistance (n = 16), supply of medicines and equipment (n = 11), training of local healthcare professionals (n = 10), provision of financial aid (n = 6), construction of medical facilities (n = 5), health education and promotion (n = 3), and immunisation (n = 1).

The 27 included Chinese articles largely paralleled the English articles in terms of publication period (from 2010 to 2022). A significant number of these articles (n = 18) focused on CMTs’ impact in multiple African countries. Six articles examined individual countries, while three articles conducted comparisons. All but one article, co-authored by researchers from Sudan and China, were published solely by Chinese researchers. About half of the articles (n = 13) were published in Chinese core journals, indicating a high quality of research.

The included Chinese articles comprised 15 reviews, 11 case studies, and 1 ethnographic study. They explored CMTs'contributions to addressing issues including essential healthcare infrastructure development (n = 17), healthcare service enhancement (n = 13), lack of healthcare workers and medicines (n = 13), health workforce development (n = 7), public health (n = 7), control of infectious diseases such as HIV/AIDS, tuberculosis, malaria, and schistosomiasis (n = 9), as well as Ebola (n = 3), COVID-19 (n = 4), and rehabilitation medicine (n = 1). Reported CMT programmes included medical team assistance (n = 20), supply of medicines and equipment (n = 18), training of local healthcare professionals (n = 16), technical cooperation and exchange (n = 16), construction of medical facilities (n = 17), public health assistance (n = 9), Chinese medicine (n = 9), short-term programmes such as the “Brightness Action” (n = 6), health education and promotion (n = 4), and immunisation (n = 1). While only English-language studies are cited in the text, an overview of the 27 Chinese studies and a summary of their key findings are provided in Supplementary Material: Appendix 5 for reference.

### Modality of CMT programmes

**Programme location**. The first CMT was deployed to Algeria in 1963, marking the beginning of CMT medical aid in Africa [11, 12, 14, 15, 23–32]. This dispatch was characterised by a “no strings attached” approach, denoting the provision of aid without any political, economic, or ideological conditions [15, 32]. By the end of 2013, 1171 CMTs had operated in 113 medical centres across 49 recipient countries, with 42 of these countries located in Africa [24, 33–38]. A significant portion of the teams served in rural areas [12, 14, 15, 31] where local medical coverage was insufficient [12]. Meanwhile, the establishment of 30 anti-malaria centres by 2013 exemplified the targeted efforts of CMTs to combat specific health challenges [28].

**Programme duration.** The duration of CMT programmes has ranged from a few months to over two years [14, 24, 26, 27, 30, 35, 36]. Short-term missions addressed immediate healthcare needs, such as during the 2014–2015 Ebola outbreak when CMTs provided treatment, infection control, and medical supplies, significantly mitigating the virus’s impact in African communities [35, 36]. Conversely, the long-term deployments aimed to foster sustainable healthcare improvements [24]. These missions, which included training local health professionals and establishing essential healthcare infrastructure such as hospitals, clinics, and malaria control centres [27], were designed to lay a lasting foundation for the ongoing enhancement of healthcare systems in recipient countries [24, 26, 27].

**Collaborative initiatives.** CMTs have collaborated with multiple local healthcare teams in Africa [12, 14, 23–25, 27, 28, 30–33, 35–38], which comprised doctors, nurses, project directors, health facility administrators, Ministry of Health officials [14, 25, 32], and representatives from non-governmental organisations [33]. The multisectoral partnerships delivered a broad range of services, from primary care to specialised treatments [14, 24, 33]. A large focus of these initiatives was on controlling and eradicating malaria [23, 24, 27, 28, 30, 31, 33, 37, 38], as well as managing other infectious diseases like measles, filariasis, schistosomiasis, HIV/AIDS, and tuberculosis [12, 27, 30, 31, 33], alongside responses to pandemics such as SARS, Ebola, and COVID-19 [12, 25, 30, 31, 35, 36]. These efforts included comprehensive strategies like vaccination campaigns and mass drug administration [12]. Meanwhile, CMTs actively tackled non-communicable diseases through short-term programmes [12, 24, 25, 33]. For instance, under the “Heart-to-Heart” programme, 170 heart surgeries were carried out in Ghana and Tanzania, while the “Brightness Action” programme facilitated 9,752 cataract surgeries in 25 countries, including Botswana, Eritrea, and Morocco [12]. Furthermore, to enhance healthcare delivery, Chinese partnerships have built hospitals, expanded local pharmaceutical production, and established over 10 clinics that integrate traditional Chinese and African medicines in remote African areas [24, 28, 31].

**Resources and expertise.** The composition of CMTs included seasoned professionals such as doctors and nurses specialised in infectious diseases, surgery, gynaecology, ophthalmology, and obstetrics [12, 14, 15, 23, 24, 26–33, 35–37], as well as public health experts [12, 35]. The teams also included translators [24, 26] and administrative support staff [30], who facilitated the smooth execution of initiatives. Provincial governments in China, such as Ningxia, Guangxi, Tianjin, and Shanghai, were responsible for deploying medical teams to specific African countries like Benin, Comoros, Gabon, and Cameroon [15, 23, 27, 30]. These teams were strategically stationed in local hospitals and clinics [37], allowing the direct allocation of resources, including medical supplies and training courses tailored to the specific needs of recipient countries [14, 24, 29, 31, 33, 36].

### Effectiveness of CMT programmes

**Meeting local healthcare needs.** Local clinics and hospitals that benefited from CMT programmes reported a reduction in patient load related to common illnesses, evidencing the effectiveness of these initiatives [12, 28, 31, 35, 38]. Campaigns like “Brightness Action” and “Heart-to-Heart” were highly praised for significantly improving specific health outcomes, particularly in rural areas [12]. Moreover, the literature demonstrates the effectiveness of CMTs in controlling infectious diseases. For example, a malaria control initiative in the Plateaux Region of Togo in 2017 reduced the infection rate from 79 to 37% [38]. In Sierra Leone, CMTs were instrumental in containing the 2014 Ebola outbreak, with no new cases two days before their departure [35]. In Zanzibar, Tanzania, CMTs provided critical assistance in schistosomiasis management, helping to reduce local infection rates [31]. Similarly, in Comoros, CMTs’ support for the malaria eradication effort through an artemisinin-based combination therapy resulted in zero deaths and a 98% reduction in the morbidity rate [12, 28, 38].

**Improving the quality of care. **Improving healthcare quality has been a key focus of CMT programmes [12, 14, 15, 28, 31, 33, 35, 38]. For instance, efforts to control malaria included not only the provision of free diagnosis and treatment but also the distribution of long-lasting insecticide-treated nets, particularly for vulnerable groups such as children and pregnant women [28, 38]. These efforts, supported by the deployment of highly skilled and dedicated professionals [14, 15], have resulted in notable improvements in both the quantity and quality of care [38]. Furthermore, China's commitment to enhancing healthcare standards across Africa is evident through the establishment of medical facilities and training institutions throughout the continent [14, 31].

**Capacity building.** The literature highlights the substantial training opportunities provided by CMTs to African health professionals, contributing to a “never-leaving” impact that reinforces local healthcare systems [12, 14, 15, 24, 25, 27, 31–38].Collaborations often extend beyond patient treatment, fostering a mutual exchange of medical knowledge and best practices. These interactions have not only facilitated the sharing of expertise but also nurtured professional respect between CMT members and local healthcare workers, ultimately improving the quality and sustainability of healthcare delivery [32, 34].

### Major drivers of CMT

**Political and diplomatic goals.** CMT programmes are crucial to China's foreign policy in Africa, often driven by political and diplomatic goals [11, 12, 14, 15, 23–25, 27–29, 32, 34, 37, 38]. The Chinese government prioritises non-interference, mutual economic development benefits, and self-determination in its engagements with African partners [11, 15, 23–25, 29, 34]. These bilateral agreements, initiated at the national level, are frequently implemented by provincial or prefectural governments [15, 27]. The Fifth International Roundtable on China-Africa Health Collaboration in March 2015 resulted in policy recommendations for Universal Health Coverage and proposed monitoring efforts [12]. Furthermore, a memorandum signed at the 2015 Forum on China-Africa Cooperation meeting in Johannesburg supported the establishment of the Africa Centres for Disease Control and Prevention, marking a significant milestone in China-Africa health cooperation [12]. According to Shen & Fan, CMTs represent not just a component of China’s foreign medical aid but also a strategic element in fostering partnerships and contributing to Africa's development goals [27].

**Economic cooperation. **CMT programmes were also motivated by economic cooperation [12, 15, 23, 24, 27–29, 31–35, 37]. China's economic ties with Africa, as manifested through medical aid, illustrated how health initiatives are integrated with broader economic and trade engagements [15, 24]. A prominent example is the 2012 Forum on China–Africa Cooperation Beijing Action Plan for 2013–2015, which aimed to scale up the China–Africa Development Fund to US$5 billion. This plan focused on enhancing technological support, sharing experiences, and boosting the capacity for independent development across African countries [27]. These efforts not only solidified the economic ties but also showed China’s commitment to addressing the health challenges in Africa [23]. Furthermore, the economic relationships helped to bolster local and national public health laboratory systems [28, 31]. For example, between 2014 and 2015, China invested in health infrastructure in Sierra Leone, including advanced laboratory facilities that enabled real-time disease surveillance and enhanced diagnostic capabilities during the Ebola outbreak [35]. As noted by Grepin et al., China's economic contributions to global health encompass more than just financial aid; they extend to providing technical support, training healthcare professionals, and developing health infrastructure [23].

**Humanitarian aid. **The literature reveals the pivotal role of humanitarian aid in CMT programmes in Africa, emphasising the human-centric approach that underpins China's commitment to raising healthcare standards [12, 14, 24, 25, 27, 28, 31, 35, 38]. Tracking back to the 1960 s, this commitment began during a period marked by China’s solidarity with newly independent African nations, advocating non-alignment and South-South cooperation [24, 25]. The deployment of medical teams, characterised by a policy of non-interference in domestic affairs, highlighted this humanitarian intent [25]. In 2015, for example, China provided substantial aid by sending five tranches of humanitarian assistance valued at US$120 million to 13 African countries, along with nearly 1,200 medical staff and public health experts [12]. Moreover, China’s collaboration with international organisations like the WHO and the United Nations reinforced its dedication to global health security and its commitment to international solidarity with Africa [12, 28, 31].

### Barriers to CMT programmes

**Infrastructure and resource disparities.** Infrastructure and resource disparities posed significant challenges to the effectiveness of CMT programmes in Africa, as evidenced by over half of the articles reviewed [12, 14, 27, 28, 30–33, 35, 36]. In many remote African communities, access to medical assistance was hindered by inadequate transportation, funding, and resources, which affected the timely delivery of essential supplies, personnel, and equipment [14, 27, 28, 30, 33, 36]. Additionally, the vast scope of health challenges—from infectious diseases to high mortality rates and poor sanitation—placed overwhelming demands on the limited resources [12, 27, 31, 35]. Daly et al., emphasised the issue of mismatched priorities in resource allocation, which impedes the sustainability of CMT programmes [32]. Notably, in Tanzania and Ghana, CMT management has faced considerable challenges in recruiting qualified doctors from their respective provinces [14].

**Cultural differences.** Several articles highlighted the significant impact of cultural differences on CMT programmes [12, 14, 32, 35, 36]. For instance, Daly et al. highlighted the professional respect between African and Chinese healthcare workers that fosters mutual learning [32]. However, challenges were also noted, with some local healthcare workers experiencing difficulties in engaging with their Chinese counterparts [12, 32]. These challenges, primarily related to cultural adjustments, communication styles, and environmental adaptations, could have affected the effectiveness of the programmes [14, 35, 36].

**Language barriers.** Language barriers constituted a significant challenge in CMT programmes across Africa [12, 14, 25, 32, 35]. While English, French, and Portuguese are widely spoken in many African countries, a substantial proportion of the population, particularly in rural areas, primarily communicates in local languages. This linguistic diversity complicates the communication of health information, treatment plans, and preventive measures [14, 32, 35]. For instance, a study conducted in Tanzania and Malawi found that African residents and Chinese healthcare workers reported strained relationships due to communication difficulties, including misunderstandings of culturally specific mannerisms, gestures, and etiquettes [32]. Such ineffective communication can hinder the successful implementation of healthcare initiatives and reduce patient trust in medical interventions [12, 25].

## Discussion

### Effectiveness of CMT programmes

This scoping review is one of the few studies to systematically examine CMTs across Africa, highlighting their unique modality, effectiveness, and primary drivers in bridging healthcare gaps in underserved areas. By synthesising both English and Chinese literature, the findings reveal that CMTs have had a substantial and diverse impact on infrastructure development, healthcare service enhancement, infectious disease control, and capacity building across the continent [24]. Deployed as early as 1963, CMTs have fostered collaborative ties with African countries and expanded their scope to include a range of essential medical services and public health initiatives [24]. The effectiveness of CMTs is evident through their provision of critical services such as malaria treatment, surgeries, and ophthalmology, which have collectively improved healthcare access and outcomes in communities that face considerable barriers to healthcare [12, 38].

### Modality of CMT programmes

CMTs stand out within international health aid due to its structured, government-led deployment and a"no strings attached"approach [15, 32]. In contrast to Western aid models, which often include conditionalities tied to political or economic reforms—as seen in the U.S. President’s Emergency Plan for AIDS Relief [39, 40], the"no strings attached"approach enables greater operational flexibility in diverse African contexts, allowing the focus to remain on local health needs without the complexities associated with conditional aid [15, 32]. This structure fosters trust and simplifies collaboration with local governments, which may otherwise be wary of aid that mandates political or economic reforms.

CMTs are often directed through provincial authorities in China, establishing a stable, long-term"twinning"approach in which Chinese provinces develop enduring partnerships with African countries [24]. This strategy, derived from China’s domestic inter-provincial support system, allows CMTs to provide consistent, well-coordinated aid over time, reinforcing relationships across both the governmental and community levels [27]. Evidence suggests that this approach has enabled greater acceptance of CMTs within local communities, particularly in rural areas where long-term engagement is essential for sustainable health improvements [15, 32].

Additionally, CMTs include a tailored mix of specialists and general practitioners deployed to address specific healthcare needs, particularly in infectious disease control, maternal and child health, and surgery [28]. Mission durations vary from short-term emergency responses to long-term engagements that can last up to two years, balancing rapid crisis response with sustained health system support [30, 32]. Importantly, CMTs integrate directly into local healthcare institutions rather than functioning as independent units, working closely with local providers to facilitate skill transfer, knowledge exchange, and capacity building [24, 35]. For instance, CMT doctors work alongside local healthcare workers during surgeries, transferring techniques that continue to benefit patients after their departure [12]. This collaboration not only fosters mutual respect but also ensures that the healthcare improvements are sustainable within the local systems [32, 35].

### Policy and implementation implications

CMTs offer significant implications for global health policy. Integrated within China’s broader diplomatic objectives, such as the Forum on China–Africa Cooperation [11, 12], they serve as a model of"South-South cooperation,"emphasising mutual respect, non-interference, and shared goals [23, 34]. Unlike many Western health aid programmes that impose political or economic conditions [39], the non-conditionality of CMT assistance gains the respect and cooperation of local governments [32], showcasing that non-conditional aid can effectively bridge healthcare gaps in resource-limited settings while respecting local governance structures.

Moreover, the flexibility and adaptability of CMTs—particularly during the Ebola and COVID-19 outbreaks—demonstrate the advantages of a government-coordinated, rapid response mechanism [12, 35]. This adaptability positions CMT programmes as a potential model for other nations seeking to enhance their global health influence. Integrating policy recommendations into CMT practices, such as continuous training and knowledge transfer for local healthcare workers, could further inform global health strategies aimed at strengthening sustainable local capacities in regions facing healthcare challenges.

### Challenges for CMTs

Despite their accomplishments, CMTs face substantial challenges that impact their effectiveness. Cultural and language barriers remain among the most significant challenges [30, 32], as CMT personnel must adapt to local customs and communicate effectively with patients and local health providers [32]. Although translators are often included [24, 26], a lack of in-depth cultural and language fluency hinders patient care and limits the effectiveness of health education campaigns [32]. Enhancing cultural competency training and expanding the role of local staff in communication roles could alleviate some of these challenges. Logistical and resource disparities also restrict CMT operations [36]. Many missions are deployed to areas with insufficient infrastructure, limited medical supplies, and inadequate transportation, complicating the delivery of consistent, high-quality care [32, 36]. Additionally, the government-mandated nature of CMT staffing means that some specialised health needs may go unmet if appropriate expertise is not available [27]. Introducing a more flexible staffing strategy that allows healthcare providers to volunteer could increase both the adaptability and impact of CMT initiatives.

### Existing literature and future research directions

The English literature on CMTs is predominantly authored by researchers from HICs, often in collaboration with Chinese scholars [14, 27, 32]. While this reflects strong international interest, the limited participation of African researchers may introduce bias in interpreting CMTs’ effectiveness. A similar pattern is observed in the Chinese literature, where only one article was co-authored by Chinese and African scholars. The current body of research is largely composed of review articles, opinion pieces, and case studies, with a notable lack of empirical studies—particularly longitudinal research—that could shed light on CMTs’ long-term impacts and operational dynamics. This scarcity restricts a comprehensive understanding of measurable outcomes and limits the research’s visibility, especially given that many studies appear in low-impact journals (Table 2).

**Table 2:** Summary of key findings from reviewed English articles

Study objectives	Emerging theme(s)	No. of articles	Reference(s)	Main findings
Modality	Programme location	20	[11, 12, 14, 15, 23–38]	The inaugural CMT was dispatched to Algeria in 1963 By 2013, 1171 CMTs had operated in 42 African countries, providing aid under a"no strings attached"policy Primarily serve rural areas, focusing on bridging healthcare gaps and combating specific diseases like malaria
	Programme duration	7	[14, 24, 26, 27, 30, 35, 36]	Ranged from a few months to over two years Short-term missions focused on immediate healthcare needs Long-term engagements aimed at sustainable healthcare improvements
	Collaborative initiatives	15	[12, 14, 23–25, 27, 28, 30–33, 35–38]	Collaborated with local healthcare teams to deliver services ranging from primary care to specialised treatments Partnerships with African countries enhanced medical service delivery to remote areas, focusing on infectious diseases and pandemic responses
	Resources and expertise	16	[12, 14, 15, 23, 24, 26–33, 35–37]	CMTs include medical professionals specialised in various fields, public health experts, and administrative staff Chinese provincial governments deploy teams, providing tailored resources and training of medical professionals
Effectiveness	Meeting local healthcare needs	5	[12, 28, 31, 35, 38]	Reduced patient load for common illnesses and improved health outcomes, particularly in rural areas Initiatives such as"Brightness Action"and"Heart-to-Heart"significantly enhanced specific health outcomes
	Improving the quality of care	8	[12, 14, 15, 28, 31, 33, 35, 38]	Raised healthcare standards in Africa by establishing medical facilities, training institutions, and targeted health initiatives Strengthened malaria control by providing free diagnosis and treatment, along with broader healthcare service enhancement
	Capacity building	14	[12, 14, 15, 24, 25, 27, 31–38]	Provided substantial training opportunities for African health professionals, ensuring a “never-leaving” impact of the Chinese medical team Extended collaborations beyond patient treatment, fostering mutual respect, knowledge exchange, and improvements in healthcare delivery
Major drivers	Political and diplomatic goals	14	[11, 12, 14, 15, 23–25, 27–29, 32, 34, 37, 38]	Driven by political and diplomatic goals, CMTs reflect China's strategic interests in Africa Used as a tool for enhancing diplomatic relations, helping to project China's influence in the region
	Economic cooperation	13	[12, 15, 23, 24, 27–29, 31–35, 37]	Economic cooperation, extending beyond healthcare, characterises CMT programmes in Africa China's contributions to global health include a variety of economic support measures, going beyond just financial aid
	Humanitarian aid	9	[12, 14, 24, 25, 27, 28, 31, 35, 38]	China's humanitarian efforts in Africa began in the 1960 s, continuing to evolve with a focus on a human-centric approach Humanitarian aid was a pivotal component of CMT programmes, underscoring China’s commitment to enhancing healthcare across the continent
Barriers	Infrastructure and resource disparities	10	[12, 14, 27, 28, 30–33, 35, 36]	Infrastructure and resource disparities impacted the timely delivery of supplies and personnel Inadequate transportation, funding, and resources limited access to medical assistance in remote African communities
	Cultural differences	5	[12, 14, 32, 35, 36]	Adjusting to different cultures, environments, and communication styles posed challenges, affecting programme effectiveness
	Language barriers	5	[12, 14, 25, 32, 35]	Language barriers posed challenges in communicating health information, treatment plans, and preventive measures Ineffective communication in local languages led to misinterpretations, hindering programme implementation

*CMT* Chinese medical team

Furthermore, while diplomatic goals, economic cooperation, and humanitarian aid were consistently cited as drivers of CMTs, none of the included studies systematically evaluated these as outcomes. Articles referenced CMTs’ role in fostering bilateral ties or facilitating broader economic engagement [23, 24], but no studies measured their direct impact on trade, diplomatic agreements, or geopolitical influence.

Future research should prioritise empirical studies, particularly those involving African scholars, to provide deeper, context-specific insights into CMTs’ contributions and limitations. Such work would help refine the design and implementation of CMT programmes, ensuring they align more effectively with local needs and contribute to more sustainable and inclusive international health cooperation. Additionally, studies should explore geopolitical and economic dimensions of CMTs, including their role in fostering diplomatic ties and economic cooperation, to provide a holistic evaluation of their impact in Africa.

### Strengths and limitations

This scoping review was strengthened by adherence to established methodological frameworks, including the Arksey and O’Malley guidelines [16] and the PRISMA-ScR checklist [17], ensuring a systematic and rigorous synthesis. The Joanna Briggs Institute Critical Appraisal Checklist further enhanced the credibility and transparency of the findings. Additionally, the inclusion of both English and Chinese sources helped minimise selection bias and ensured a more comprehensive representation of diverse cultural perspectives.

However, certain limitations should be noted. The reliance on peer-reviewed articles may have introduced publication bias, potentially overrepresenting studies with favourable outcomes [41]. Additionally, studies published after February 2023 were not included, meaning recent developments in CMT programmes may not be reflected. Furthermore, this review focused on programme modalities and effectiveness, it did not examine broader financial trends in China’s health-related aid. This gap limits a fuller understanding of CMTs'financial sustainability, resource allocation, and integration within China's global health strategy. While the inclusion of authors with direct experience in institutions collaborating with CMTs provides valuable insights, it is acknowledged that their perspectives are shaped by their affiliations and academic backgrounds. This underscores the importance of continued dialogue and collaboration across diverse disciplines and regions to ensure a more comprehensive evaluation of CMTs'role in African healthcare systems.

## Conclusions

This review underscores the vital role of CMTs in addressing healthcare disparities in Africa through a distinctive, non-conditional, and government-led approach. Their contributions to healthcare system strengthening, emergency response, and capacity-building demonstrate their importance in global health initiatives. CMTs’ adaptability, particularly in crises like Ebola and COVID-19, and their integration within local systems further highlight their potential for fostering sustainable international health collaborations. However, their effectiveness is moderated by structural and operational challenges, including resource limitations, cultural and language barriers, and sustainability concerns. These factors may constrain the long-term impact of CMTs, underscoring the need for more flexible staffing models, enhanced cultural training, and stronger integration with local healthcare systems.

## Supplementary Information

Additional file 1

## Data Availability

The case information sources are listed in Supplementary Material: Appendix, and all data and materials used can be accessed from the corresponding author upon reasonable request.

## Abbreviations

AIDS: Acquired immunodeficiency syndrome
CMT: Chinese medical team
COVID-19: Coronavirus disease
DAH: Development assistance for health
FOCAC: The forum on China–Africa cooperation
HICs: High-income countries
HIV: Human immunodeficiency virus
IF: Impact factor
LMICs: Low-and middle-income countries
PRISMA-ScR: Preferred reporting items for systematic reviews and meta-analyses extension for scoping reviews
SARS: Severe acute respiratory syndrome
WHO: World Health Organization
