# Supplementary material for: The role of Chinese medical teams in bridging healthcare gaps in Africa: a scoping review
Source: Glob Health Res Policy. 2025 Jun 16;10:23. doi: 10.1186/s41256-025-00420-2 (PMC12168301; doi:10.1186/s41256-025-00420-2)
Supplement: Supplementary file 1 — Additional file 1 [file 41256_2025_420_MOESM1_ESM.docx]

**Supplementary Material**

**Appendix**

**Contents**

[Appendix 1: The Role of Chinese Medical Teams in Bridging Healthcare Gaps in Africa: A Scoping Review Protocol 1](#_Toc170247488)

[Appendix 2: Preferred Reporting Items for Systematic reviews and Meta-Analyses extension for Scoping Reviews (PRISMA-ScR) Checklist 4](#_Toc170247489)

[Appendix 3: Search Strategy and Results 6](#_Toc170247490)

[Appendix 4: JBI Critical Appraisal Checklist 9](#_Toc170247491)

[Appendix 5: Characteristics of the 27 Chinese studies included for evidence synthesis and analysis 12](#_Toc170247492)

# **Appendix 1:** The Role of Chinese Medical Teams in Bridging Healthcare Gaps in Africa: A Scoping Review Protocol

**Background**

Access to healthcare is a basic human right, but it remains a significant challenge in many sub-Saharan African countries. Sub-Saharan Africa is struggling to achieve universal health coverage (UHC) by 2030, and economic constraints and inadequate healthcare infrastructure are major contributors to this challenge. China provides medical aid to Africa by deploying medical teams called Chinese medical teams (CMTs). CMTs have served over 180 million patients across 51 African countries since 1963, providing on-the-ground medical support, sharing expertise, and training local healthcare workers. This scoping review aims to consolidate existing literature on the impact of CMTs in mitigating healthcare access disparities in African countries and identify the primary drivers and barriers to their activities. The findings of this review will provide valuable insights into the role and effectiveness of CMTs, supporting strategic planning and policy-making regarding China's foreign medical aid and identifying research gaps that warrant further exploration.

**Central question**

To describe and systematically map existing literature on CMTs’ efforts in bridging the healthcare gaps in Africa; synthesise the research about the modality and effectiveness of CMTs, including identifying the major drivers and barriers of CMTs and the knowledge gaps that may usefully be addressed by future research.

**Review objectives**

To better explain CMT programmes in addressing local healthcare needs in Africa, the review will specifically examine the following;

1. The modality and effectiveness of CMT programmes in Africa.

2. The primary drivers and barriers for CMTs in their efforts to bridge local healthcare gaps.

**Review Methods**

Overview & Justification

We plan to use the scoping review methodology, as described in the work of Arksey and O'Malley, to analyse both qualitative and quantitative evidence on the activities of CMTs within the healthcare of African countries. Our study protocol will follow the Preferred Reporting Items for Systematic Reviews and Meta-Analyses (PRISMA) extension for the scoping reviews checklist. Scoping reviews are useful for “identifying the extent and range of research available on a particular subject, offering a clear picture of the amount of existing literature and the focus of the studies. They are particularly valuable for exploring new evidence in areas where it is not yet clear what specific questions should be addressed by a more detailed systematic review”.

Inclusion and exclusion criteria

We will include studies published in English and Chinese that provide qualitative and quantitative evidence on the impact of CMTs in Africa. We will specifically target publications detailing the modality and effectiveness of CMT programmes and exploring the drivers and barriers these programmes encounter in bridging local healthcare gaps. This will be developed based on the selection criteria by the Population, Concept, Context (PCC) framework. We will exclude studies not directly related to health aid, general discussions about China’s medical aid to Africa without a specific focus on CMTs, conference proceedings, commentaries, abstracts, book reviews, and papers for which full texts were not accessible.

-Research detail:

a) Languages: English and Chinese;

b) Published Time: Inception- Feb. 2023;

c) Qualitative and quantitative evidence on the impact of CMTs in Africa;

d) Publications detailing the modality and effectiveness of CMT programmes and

e) Drivers and barriers these programmes encounter in bridging local healthcare gaps

Search strategy

- Database

a) English databases: PubMed, Web of Science (WoS), ScienceDirect, Scopus, Cumulative Index to Nursing and Allied Health Literature (CINAHL);

b) Chinese databases: China National Knowledge Infrastructure and Wanfang Database.

-Search keyword

Free-text terms ("medical assistance" OR "medical team" OR "medical program" OR "bilateral assistance" OR "bilateral support" OR "bilateral funding" OR "bilateral aid" OR "CMT" OR "CMA" OR "foreign aid" OR "foreign assistance" OR "medical mission" OR "medical group" OR "medical intervention" OR "medical service") AND (China OR Chinese) AND (Africa OR "low-and middle-income countries" OR "LMICs"). We first tested the search string in PubMed and, after validating, will be adapted to other databases.

-Search steps

The reviewers will use inclusion and exclusion criteria to screen all titles and abstracts of retrieved studies for eligibility. If an article potentially meets the criteria, two reviewers will independently evaluate the full text. Any discrepancies will be discussed, and if a consensus cannot be reached, a third reviewer will be brought in to mediate.

**Data extraction**

We will follow the PRISMA guidelines to conduct the study. Data from the included studies will be extracted by one reviewer and then checked by a second reviewer. We will extract essential information from the studies, and the data extraction table will be provided at a later stage.

# Appendix 2: Preferred Reporting Items for Systematic reviews and Meta-Analyses extension for Scoping Reviews (PRISMA-ScR) Checklist

| **SECTION** | **ITEM** | **PRISMA-ScR CHECKLIST ITEM** | **REPORTED ON**  **PAGE #** |
| --- | --- | --- | --- |
| **TITLE** | | | |
| Title | 1 | Identify the report as a scoping review. | 1 |
| **ABSTRACT** | | | |
| Structured summary | 2 | Provide a structured summary that includes (as applicable): background, objectives, eligibility criteria, sources of evidence, charting methods, results, and conclusions that relate to the review questions and objectives. | 2 |
| **INTRODUCTION** | | | |
| Rationale | 3 | Describe the rationale for the review in the context of what is already known. Explain why the review questions/objectives lend themselves to a scoping review approach. | 3-4 |
| Objectives | 4 | Provide an explicit statement of the questions and objectives being addressed with reference to their key elements (e.g., population or participants, concepts, and context) or other relevant key elements used to conceptualize the review questions and/or objectives. | 4 |
| **METHODS** | | | |
| Protocol and registration | 5 | Indicate whether a review protocol exists; state if and where it can be accessed (e.g., a Web address); and if available, provide registration information, including the registration number. | Appendix 1 |
| Eligibility criteria | 6 | Specify characteristics of the sources of evidence used as eligibility criteria (e.g., years considered, language, and publication status), and provide a rationale. | 5 |
| Information sources* | 7 | Describe all information sources in the search (e.g., databases with dates of coverage and contact with authors to identify additional sources), as well as the date the most recent search was executed. | 5 & Appendix 3 |
| Search | 8 | Present the full electronic search strategy for at least 1 database, including any limits used, such that it could be repeated. | 5 & Appendix 3 |
| Selection of sources of evidence† | 9 | State the process for selecting sources of evidence (i.e., screening and eligibility) included in the scoping review. | 5 |
| Data charting process‡ | 10 | Describe the methods of charting data from the included sources of evidence (e.g., calibrated forms or forms that have been tested by the team before their use, and whether data charting was done independently or in duplicate) and any processes for obtaining and confirming data from investigators. | 6 |
| Data items | 11 | List and define all variables for which data were sought and any assumptions and simplifications made. | 6 |
| Critical appraisal of individual sources of evidence§ | 12 | If done, provide a rationale for conducting a critical appraisal of included sources of evidence; describe the methods used and how this information was used in any data synthesis (if appropriate). | 6 & Appendix 4 |
| Synthesis of results | 13 | Describe the methods of handling and summarizing the data that were charted. | 7 |
| **RESULTS** | | | |
| Selection of sources of evidence† | 14 | Give number of sources of evidence screened, assessed for eligibility, and included in the review, with reasons for exclusions at each stage, ideally using a flow diagram. | 7 & Figure 1 |
| Characteristics of sources of evidence | 15 | For each source of evidence, present characteristics for which data were charted and provide the citations. | 8-10 |
| Critical appraisal within sources of evidence | 16 | If done, present data on critical appraisal of included sources of evidence (see item 12). | 6 & Appendix 4 |
| Results of individual sources of evidence | 17 | For each included source of evidence, present the relevant data that were charted that relate to the review questions and objectives. | 10-17 |
| Synthesis of results | 18 | Summarize and/or present the charting results as they relate to the review questions and objectives. | 8-17 |
| **DISCUSSION** | | | |
| Summary of evidence | 19 | Summarize the main results (including an overview of concepts, themes, and types of evidence available), link to the review questions and objectives, and consider the relevance to key groups. | 17-22 |
| Limitations | 20 | Discuss the limitations of the scoping review process. | 22 |
| Conclusions | 21 | Provide a general interpretation of the results with respect to the review questions and objectives, as well as potential implications and/or next steps. | 22 |
| **FUNDING** | | | |
| Funding | 22 | Describe sources of funding for the included sources of evidence, as well as sources of funding for the scoping review. Describe the role of the funders of the scoping review. | 23 |

JBI = Joanna Briggs Institute; PRISMA-ScR = Preferred Reporting Items for Systematic reviews and Meta-Analyses extension for Scoping Reviews.

* Where *sources of evidence* (see second footnote) are compiled from, such as bibliographic databases, social media

platforms, and Web sites.

† A more inclusive/heterogeneous term used to account for the different types of evidence or data sources (e.g., quantitative and/or qualitative research, expert opinion, and policy documents) that may be eligible in a scoping review as opposed to only studies. This is not confused with *information sources* (see first footnote).

‡ The frameworks by Arksey and O’Malley (6) and Levac and colleagues (7) and the JBI guidance (4, 5) refer to the process of data extraction in a scoping review as data charting*.*

§ The process of systematically examining research evidence to assess its validity, results, and relevance before using it to inform a decision. This term is used for items 12 and 19 instead of "risk of bias" (which is more applicable to systematic reviews of interventions) to include and acknowledge the various sources of evidence that may be used in a scoping review (e.g., quantitative and/or qualitative research, expert opinion, and policy document).

*From:* Tricco AC, Lillie E, Zarin W, O'Brien KK, Colquhoun H, Levac D, et al. PRISMA Extension for Scoping Reviews (PRISMAScR): Checklist and Explanation. Ann Intern Med.

# Appendix 3: Search Strategy and Results

**Table 1: PubMed search strategy** (PubMed, 21/02/2023 03:33:22) **(n=106)**

| **Search number #** | **Search Query** | **Results** |
| --- | --- | --- |
| #1 | "medical assistance" OR "medical team" OR "medical program" OR "bilateral assistance" OR "bilateral support" OR "bilateral funding" OR "bilateral aid" OR "CMT" OR "CMA" OR "foreign aid" OR "foreign assistance" OR "medical mission" OR "medical group" OR "medical intervention" OR "medical service" | 70,075 |
| #2 | China OR Chinese | 2,801,815 |
| #3 | Africa OR "Low-and middle-income countries" OR "LMICs" | 468,213 |
|  | **(#1) AND (#2) AND (#3)** | **106** |

**Table 2: Web of Science search strategy** (Tuesday, February 21/02/ 2023 17:33:00 GMT+0800) (China Standard Time) **(n=97)**

| **Search Category #** | **Search Query** | **Results** |
| --- | --- | --- |
| #1 | TS= (China OR Chinese) | 1248816 |
| #2 | TS= ("medical assistance" OR "medical team" OR "medical program" OR "bilateral assistance" OR "bilateral support" OR "bilateral funding" OR "bilateral aid" OR "CMT" OR "CMA" OR "foreign aid" OR "foreign assistance" OR "medical mission" OR "medical group" OR "medical intervention" OR "medical service") | 65199 |
| #3 | TS= (Africa OR "Low- and middle-income countries" OR "LMICs") | 594211 |
|  | **(#1) AND (#2) AND (#3)** | **97** |

**Table 3: Scopus search strategy** (Wednesday, February 22/02/ 2023 08:35:00 GMT+0800) (China Standard Time) **(n=232)**

| **Item** | **Search Query** | **Results** |
| --- | --- | --- |
| 1 | ALL (China OR Chinese) | 15,223,830 |
| 2 | ALL (medical AND assistance OR medical AND team OR medical AND program OR bilateral AND assistance OR bilateral AND support OR bilateral AND funding OR bilateral AND aid OR CMT OR CMA OR foreign AND aid OR foreign AND assistance OR medical AND mission OR medical AND group OR medical AND intervention OR medical AND service) | 11,040 |
| 3 | ALL (Africa OR low-and middle-income countries OR LMICs) | 174,942 |
|  | **1 AND 2 AND 3** | **232** |

**Table 4: Cumulative Index to Nursing and Allied Health Literature search strategy** (Tuesday, February 22/02/ 2023 20:35:00 GMT+0800) (China Standard Time) **(n=96)**

| **Search ID #** | **Search Query** | **Actions** |
| --- | --- | --- |
| S1 | TX ((China OR Chinese)) | (105,900) |
| S2 | TX (("medical assistance" OR "medical team" OR "medical program" OR "bilateral assistance" OR "bilateral support" OR "bilateral funding" OR "bilateral aid" OR "CMT" OR "CMA" OR "foreign aid" OR "foreign assistance" OR "medical mission" OR "medical group" OR "medical intervention" OR "medical service")) | (10,343) |
| S3 | TX ((Africa OR "low-and middle-income countries" OR "LMICs")) | (51,554) |
|  | **S1 AND S2 AND S3** | **96** |

**Table 4: ScienceDirect search strategy** (Tuesday, February 22/02/ 2023 21:15:00 GMT+0800) (China Standard Time) **(n=397)**

| **Search ID #** | **Query** | **Actions** |
| --- | --- | --- |
| ALL | ("medical assistance" OR "medical team" OR "medical program" OR "bilateral assistance" OR "bilateral support" OR "bilateral funding" OR "bilateral aid" OR "CMT" OR "CMA" OR "foreign aid" OR "foreign assistance" OR "medical mission" OR "medical group" OR "medical intervention" OR "medical service") AND (China OR Chinese) AND (Africa OR "Low-and middle-income countries" OR "LMICs") | **397** |

**Table 5: CNKI search strategy** **(n=615)**

| **Item #** | **Query** | **Actions** |
| --- | --- | --- |
| ALL | ("援非医疗队" OR "援外医疗队" OR "医疗援助" OR "医疗外交" OR "卫生外交" OR "对外援助") AND ("非洲" OR "中非" OR "中非合作" OR "南南合作") | **615** |

**Table 6: Wanfang search strategy** **(n=44)**

| **Item #** | **Query** | **Actions** |
| --- | --- | --- |
| ALL | ("援非医疗队" OR "援外医疗队" OR "医疗援助" OR "医疗外交" OR "卫生外交" OR "对外援助") AND ("非洲" OR "中非" OR "中非合作" OR "南南合作") | **441** |

# Appendix 4: JBI Critical Appraisal Checklist

**Table 1** JBI Critical Appraisal Checklist for textual evidence: Narrative in 12 studies on CMT programmes in Africa

| Studies | Is the generator of the narrative a credible or appropriate source? | Is the relationship between the text and its context explained? (where, when, who with, how) | Does the narrative present the events using a logical sequence so the reader or listener can understand how it unfolds? | Do you, as reader or listener of the narrative, arrive at similar conclusions to those drawn by the narrator? | Do the conclusions flow from the narrative account? | Do you consider this account to be a narrative? |
| --- | --- | --- | --- | --- | --- | --- |
| Wang. et al. | Yes | Yes | Yes | Yes | Yes | Yes |
| Liu. et al. | Yes | Yes | Yes | Yes | Yes | Yes |
| Chen. et al. | Yes | Yes | Yes | Yes | Yes | Yes |
| Shen & Fan | Yes | Yes | Yes | Unclear | Yes | Yes |
| Cheng & Shi | Yes | Yes | Yes | Unclear | Yes | Yes |
| Yanzhong | Yes | Yes | Yes | Unclear | Yes | Yes |
| Lin. et al. | Yes | Unclear | Yes | N/A | Yes | Yes |
| Tambo. et al. | Yes | Unclear | Yes | N/A | Unclear | Yes |
| Samy | Yes | Unclear | Yes | N/A | Yes | Yes |
| Morgan & Zheng | Yes | Yes | Yes | N/A | Yes | Yes |
| Wang & Sun | Yes | Unclear | Yes | N/A | Unclear | Yes |
| Xia. et al. | Yes | Unclear | Yes | Yes | Yes | Yes |

**Table 2** JBI Critical Appraisal Checklist for qualitative research in 5 studies in CMT programmes in Africa

| Studies | Is there congruity between the stated philosophical  perspective and the research methodology? | Is there congruity between the research methodology and the research question or objectives? | Is there congruity between the research methodology and the methods used to collect data? | Is there congruity between the research methodology and the representation and analysis of data? | Is there congruity between the research methodology and the interpretation of results? | Are participants, and their voices, adequately represented? | Is the research ethical according to current criteria or, for recent studies, and is there evidence of ethical approval by an appropriate body? | Do the conclusions drawn in the research report flow from the analysis, or interpretation, of the data? |
| --- | --- | --- | --- | --- | --- | --- | --- | --- |
| Li. et al. | Yes | Yes | Yes | Yes | Yes | Yes | Yes | Yes |
| Daly. et al. | Yes | Yes | Yes | Yes | Yes | Yes | Yes | Yes |
| Chen. et al. | Yes | Yes | Yes | Yes | Yes | Yes | Yes | Yes |
| Zhou & Ma | Unclear | Yes | Yes | Yes | Yes | Unclear | Unclear | Yes |
| Lu. et al. | Yes | Yes | Yes | Yes | Yes | Unclear | N/A | Yes |

**Table 3** JBI Critical Appraisal Checklist for analytical cross-sectional studies in 3 studies in CMT programmes in Africa

| Studies | Were the criteria for inclusion in the sample clearly defined? | Were the study subjects and the setting described in detail? | Was the exposure measured in a valid and reliable way? | Were objective, standard criteria used for measurement of the condition? | Were confounding factors identified? | Were strategies to deal with confounding factors stated? | Were the outcomes measured in a valid and reliable way? | Was appropriate statistical analysis used? |
| --- | --- | --- | --- | --- | --- | --- | --- | --- |
| Grepin. et al. | Yes | Yes | Unclear | Unclear | Yes | Yes | Unclear | Yes |
| Yang. et al. | Yes | Yes | Unclear | Yes | Yes | Unclear | Yes | Yes |
| Shajalal. et al. | Yes | Yes | Unclear | Yes | Yes | No | Yes | Yes |

# Appendix 5:

**Table 4** Characteristics of the 27 Chinese studies included for evidence synthesis and analysis

| **编号**  **No.** | **篇名**  **Title** | **发表年份**  **Year of publication** | **作者**  **Author** | **期刊**  **Journal** | **是否核心**  **Chinese Core journal (Yes/No)** | **文章类型**  **Article type and study design** | **目标国家**  **Targeted country** | **目标问题**  **Targeted issue** | **干预措施**  **CMT Intervention** |
| --- | --- | --- | --- | --- | --- | --- | --- | --- | --- |
| 1 | 我国对外医疗援助50年回顾与思考 A Review and Reflection on 50 Years of China’s Foreign Medical Aid | 2010 | 张善纲，浦金辉，赵育新，姚国庆，杨铭，谢 峻，李达 | 人民军医 People’s Military Surgeon | 否 No | 综述  Review | 多个非洲国家 Multiple African countries | 医疗基础设施改善 Essential healthcare infrastructure development  缺医少药 Lack of healthcare workers and medicines | 援建医疗卫生机构 Construction of medical facilities  培训当地医务人员 Training of local healthcare professionals  技术合作与交流 Technical cooperation and exchange  提供药品和医疗设备 Supply of medicines and equipment |
| 2 | 江苏省援外医疗发展回顾与建议——基于援马耳他与桑给巴尔医疗队情况比较 Review and Suggestions on Jiangsu Province’s Foreign Medical Development — A Comparative Study of Medical Teams in Malta and Zanzibar | 2013 | 李波，巴璐，朱立国，戎彧 | 中华灾害救援医学 Chinese Journal of Disaster Medicine and Rescue | 否 No | 案例研究 Case study | 马耳他，桑吉巴尔 Malta, Zanzibar | 卫生事业发展 Healthcare service enhancement  缺医少药 Lack of healthcare workers and medicines | 派遣医疗队 Medical team assistance  提供药品和医疗设备 Supply of medicines and equipment  援建医疗卫生机构 Construction of medical facilities  技术合作与交流 Technical cooperation and exchange  中医药 Chinese medicine |
| 3 | 新冠肺炎全球大流行下非洲的抗疫和中非合作 Africa’s Pandemic Response and China-Africa Cooperation under the Global COVID-19 Pandemic | 2013 | 刘海方 | 国际政治研究 International Politics Studies | 是 Yes | 综述 Review | 多个非洲国家 Multiple African countries | 新冠疫情 Covid-19  公共卫生体系建设 Public health | 公共卫生援助 Public health assistance  健康教育和健康促进 Health education and promotion  援建医疗卫生机构 Construction of medical facilities  技术合作与交流 Technical cooperation and exchange |
| 4 | 湖南对非医疗援助的历史、现状及反思 The History, Status, and Reflection on Hunan’s Medical Aid to Africa | 2013 | 李明磊 | 南方论刊 Southern Journal | 否 No | 案例研究 Case study | 塞拉利昂，津巴布韦 Sierra Leone, Zimbabwe | 卫生事业发展 Healthcare service enhancement  缺医少药 Lack of healthcare workers and medicines  卫生人力资源开发 Health workforce development | 派遣医疗队 Medical team assistance  提供药品和医疗设备 Supply of medicines and equipment  中医药 Chinese medicine |
| 5 | 早期中国医疗队在非洲（1963~1978） The Early Chinese Medical Teams in Africa (1963–1978) | 2013 | 丁旭虹，张大庆 | 医学与哲学 Medicine and Philosophy | 是 Yes | 综述 Review | 多个非洲国家 Multiple African countries | 卫生事业发展 Healthcare service enhancement  缺医少药 Lack of healthcare workers and medicines  卫生人力资源开发 Health workforce development | 派遣医疗队 Medical team assistance  提供药品和医疗设备 Supply of medicines and equipment  中医药 Chinese medicine |
| 6 | 应对埃博拉与中国医疗援助模式的转型 Responding to Ebola and the Transformation of China’s Medical Aid Model | 2014 | 陈佳骏 | 国际关系研究 International Relations Studies | 否 No | 综述 Review | 多个非洲国家 Multiple African countries | 抗击埃博拉 Ebola  公共卫生体系建设 Public health | 援建医疗卫生机构 Construction of medical facilities  提供药品和医疗设备 Supply of medicines and equipment  派遣医疗队 Medical team assistance  培训当地医务人员 Training of local healthcare professionals  公共卫生援助 Public health assistance  “光明行”等短期项目 Short-term programmes like the “Brightness Action” |
| 7 | 援外医疗工作中构建医患共同体的实践及思考 Practice and Reflections on Building a Doctor-Patient Community in Foreign Medical Aid Work | 2014 | 田娟，李小刚，何岚 | 中国医学伦理学 Chinese Medical Ethics | 是 Yes | 案例研究 Case study | 马拉维 Malawi | 卫生事业发展 Healthcare service enhancement  缺医少药 Lack of healthcare workers and medicines  卫生人力资源开发 Health workforce development | 派遣医疗队 Medical team assistance  培训当地医疗人员 Training of local healthcare professionals  健康教育和健康促进 Health education and promotion |
| 8 | 后埃博拉时期中非卫生合作的趋向、挑战与建议 Trends, Challenges, and Suggestions for China-Africa Health Cooperation in the Post-Ebola Era | 2014 | 郭佳 | 国际展望 International Outlook | 是 Yes | 综述 Review | 多个非洲国家 Multiple African countries | 抗击埃博拉 Ebola  血吸虫病防治 Schistosomiasis prevention  公共卫生体系建设 Public health  疟疾防控 Malaria control | 援建医疗卫生机构 Construction of medical facilities  派遣医疗队 Medical team assistance  提供药品和医疗设备 Supply of medicines and equipment  培训当地医务人员 Training of local healthcare professionals  公共卫生援助 Public health assistance  “光明行”等短期项目 Short-term programmes like the “Brightness Action”  技术合作与交流 Technical cooperation and exchange  中医药 Chinese medicine |
| 9 | 中国与苏丹卫生合作的发展历程及主要成就 The Development and Achievements of China–Sudan Health Cooperation | 2015 | 加法尔·卡拉尔·艾哈迈德（苏丹 Sudan），王广大 | 阿拉伯世界研究 Arab World Studies | 是 Yes | 案例研究 Case study | 苏丹 Sudan | 卫生事业发展 Healthcare service enhancement  缺医少药 Lack of healthcare workers and medicines  疟疾控制  Malaria control  新冠疫情 Covie-19  公共卫生体系建设 Public health | 援建医疗卫生机构 Construction of medical facilities  派遣医疗队 Medical team assistance  提供药品和医疗设备 Supply of medicines and equipment  公共卫生援助 Public health assistance  “光明行”等短期项目 Short-term programmes like the “Brightness Action”  培训当地医务人员 Training of local healthcare professionals  技术合作与交流 Technical cooperation and exchange |
| 10 | 行动者视角：援非医疗队制度与实践的边界 From the Actor’s Perspective: Boundaries of the Chinese Medical Team System and Practice in Africa | 2015 | 高良敏 | 中山大学学报（社会科学版） Journal of Sun Yat-sen University (Social Science Edition) | 是 Yes | 民族志研究 Ethnography | 多个非洲国家 Multiple African countries |  | 培训当地医疗人员 Training of local healthcare professionals  健康教育和健康促进 Health education and promotion  技术合作与交流 Technical cooperation and exchange  中医药 Chinese medicine |
| 11 | 新时期援非医疗工作的挑战与对策 Challenges and Countermeasures in New-Era Medical Work in Africa | 2015 | 黄毓 | 现代医院 Modern Hospital | 否 No | 综述 Review | 多个非洲国家 Multiple African countries | 医疗基础设施改善 Essential healthcare infrastructure development  卫生事业发展 Healthcare service enhancement  缺医少药 Lack of healthcare workers and medicines | 援建医疗卫生机构 Construction of medical facilities  派遣医疗队 Medical team assistance  提供药品和医疗设备 Supply of medicines and equipment  培训当地医疗人员 Training of local healthcare  技术合作与交流 Technical cooperation and exchange |
| 12 | 中国援外医疗技术合作组国外工作开展情况简述 A Brief Overview of China’s Foreign Medical Technology Cooperation Teams | 2017 | 亓官晨 | 基础医学与调查报告 Basic Medicine and Investigation Reports | 否 No | 综述 Review | 多个非洲国家 Multiple African countries | 卫生事业发展 Healthcare service enhancement  缺医少药 Lack of healthcare workers and medicines  传染病防控 Control of infectious diseases | 派遣医疗队 Medical team assistance  援建医疗卫生机构 Construction of medical facilities  提供药品和医疗设备 Supply of medicines and equipment  培训当地医务人员 Training of local healthcare professionals  技术合作与交流 Technical cooperation and exchange |
| 13 | 非洲康复医学发展与中国援非医疗队 The Development of Rehabilitation Medicine in Africa and the Role of China’s Medical Teams | 2018 | 张善纲，樊光辉 | 中国康复理论与实践 Chinese Journal of Rehabilitation Theory and Practice | 是 Yes | 综述 Review | 多个非洲国家 Multiple African countries | 康复医学 Rehabilitation medicine | 派遣医疗队 Medical team assistance  提供药品和医疗设备 Supply of medicines and equipment  培训当地医务人员 Training of local healthcare professionals |
| 14 | 在非洲种下中医的种子 Planting the Seeds of Traditional Chinese Medicine in Africa | 2018 | 郑喜，郭亚东 | 中国投资 China Investment | 否 No | 案例研究 Case article | 苏丹 Sudan | 卫生事业发展 Healthcare service enhancement  缺医少药 Lack of healthcare workers and medicines  疟疾防控 Malaria control  新冠疫情 Covid-19 | 派遣医疗队 Medical team assistance  中医药 Chinese medicine |
| 15 | 新冠肺炎疫情促中非医疗卫生合作 COVID-19 Promotes China–Africa Health Cooperation | 2018 | 曾爱平 | 中国投资 China Investment | 否 No | 综述 Review | 多个非洲国家 Multiple African countries | 医疗基础设施改善 Essential healthcare infrastructure development  疟疾防控 Malaria control  新冠疫情 Covid-19  抗击埃博拉 Ebola  传染病防控 Control of infectious diseases  公共卫生体系建设 Public health | 援建医疗卫生机构 Construction of medical facilities  派遣医疗队 Medical team assistance  提供药品和医疗设备 Supply of medicines and equipment  培训当地医务人员 Training of local healthcare professionals  公共卫生援助 Public health assistance  技术合作与交流 Technical cooperation and exchange |
| 16 | 援非医疗队项目实践路径的创新与探索——以四川大学华西医院为例 Innovations and Exploration in the Practice of Medical Aid Projects in Africa — A Case Study of West China Hospital of Sichuan University | 2019 | 曾波 | 综合研究 Comprehensive Research | 否 No | 案例研究Case article | 圣多美和普林西比，莫桑比克 São Tomé and Príncipe and Mozambique | 医疗基础设施改善 Essential healthcare infrastructure development  缺医少药 Lack of healthcare workers and medicines | 派遣医疗队 Medical team assistance  提供药品和医疗设备 Supply of medicines and equipment  援建医疗卫生机构 Construction of medical facilities  培训当地医务人员 Training of local healthcare professionals |
| 17 | 中国和毛里塔尼亚医疗卫生合作的现状与未来 The Present and Future of China–Mauritania Medical and Health Cooperation | 2020 | 胡美 | 河北科技大学学报（社会科学版） Journal of Hebei University of Science and Technology (Social Science Edition) | 否 No | 案例研究 Case article | 毛里塔尼亚 Mauritania | 医疗基础设施改善 Essential healthcare infrastructure development  卫生事业发展 Healthcare service enhancement  缺医少药 Lack of healthcare workers and medicines  卫生人力资源开发 Health workforce development  公共卫生体系建设 Public health | 派遣医疗队 Medical team assistance  援建医疗卫生机构 Construction of medical facilities  培训当地医务人员 Training of local healthcare professionals  技术合作与交流 Technical cooperation and exchange  “光明行”等短期项目 Short-term programmes like the “Brightness Action” |
| 18 | 中国对喀麦隆的医疗援助：内容与成效调研 Medical Aid to Cameroon: Content and Effectiveness Evaluation | 2020 | 周海金 | 国际论坛 International Forum | 是 Yes | 案例研究Case article | 喀麦隆 Cameroon | 医疗基础设施改善 Essential healthcare infrastructure development  卫生事业发展 Healthcare service enhancement  缺医少药 Lack of healthcare workers and medicines  卫生人力资源开发 Health workforce development  传染病防控 Control of infectious diseases | 派遣医疗队 Medical team assistance  援建医疗卫生机构 Construction of medical facilities  提供药品和医疗设备 Supply of medicines and equipment  培训当地医务人员 Training of local healthcare professionals  技术合作与交流 Technical cooperation and exchange  中医药 Chinese medicine |
| 19 | 全球治理视角下的中国对非洲医疗援助 China’s Medical Aid to Africa from a Global Governance Perspective | 2020 | 高涵柏 | 市场周刊 Market Weekly | 否 No | 综述 Review | 多个非洲国家 Multiple African countries | 医疗基础设施改善 Essential healthcare infrastructure development  传染病防控 Control of infectious diseases | 派遣医疗队 Medical team assistance  援建医疗卫生机构 Construction of medical facilities  提供药品和医疗设备 Supply of medicines and equipment  公共卫生援助 Public health assistance  中医药 Chinese medicine |
| 20 | 对外医疗援助的新思维 New Thinking on Foreign Medical Aid | 2020 | 张善纲，浦金辉，赵育新，杨铭，谢峻 | 解放军医院管理杂志 Journal of Hospital Administration of the People's Liberation Army | 是 Yes | 综述 Review | 多个非洲国家 Multiple African countries |  |  |
| 21 | 我国对外医疗援助的新态势 New Trends in China’s Foreign Medical Aid | 2021 | 张善纲，赵育新，姚国庆，杨铭，谢峻，李达 | 华南国防医学杂志 South China Journal of National Defense Medicine | 是 Yes | 综述 Review | 多个非洲国家 Multiple African countries |  |  |
| 22 | 新时期对外医疗援助的意义及应对 The Significance and Response Strategies of Foreign Medical Aid in the New Era | 2021 | 张善纲，赵育新，姚国庆，杨铭，祝松，谢峻 | 解放军医院管理杂志 Journal of Hospital Administration of the People's Liberation Army | 是 Yes | 综述 Review | 多个非洲国家 Multiple African countries |  |  |
| 23 | 关于援非医疗模式的思考 Reflections on the Aid Model of China’s Medical Teams in Africa | 2021 | 宋晓风，郭亚东 | 人口与健康 Population and Health | 否 No | 综述 Review | 多个非洲国家 Multiple African countries |  | 派遣医疗队 Medical team assistance  援建医疗卫生机构 Construction of medical facilities  提供药品和医疗设备 Supply of medicines and equipment  培训当地医务人员 Training of local healthcare professionals  技术合作与交流 Technical cooperation and exchange  “光明行”等短期项目 Short-term programmes like the “Brightness Action”  中医药 Chinese medicine |
| 24 | 中非健康卫生合作“授人以渔” China–Africa Health Cooperation: “Teaching One to Fish” | 2022 | 王云屏 | 中国投资 China Investment | 否 No | 综述 Review | 多个非洲国家 Multiple African countries | 基础医疗设施改善 Essential healthcare infrastructure development  卫生人力资源开发 Health workforce development | 派遣医疗队 Medical team assistance  援建医疗卫生机构 Construction of medical facilities  提供药品和医疗设备 Supply of medicines and equipment  技术合作与交流 Technical cooperation and exchange  公共卫生援助 Public health assistance  “光明行”等短期项目 Short-term programmes like the “Brightness Action” |
| 25 | 中非卫生合作的特点：基于刚果民主共和国的案例研究 Characteristics of China–Africa Health Cooperation: A Case Study of the Democratic Republic of Congo | 2022 | 蒋晓晓 | 中国卫生政策研究 Chinese Journal of Health Policy | 是 Yes | 案例研究Case article | 刚果民主共和国 The Democratic Republic of Congo |  | 派遣医疗队 Medical team assistance  援建医疗卫生机构 Construction of medical facilities  提供药品和医疗设备 Supply of medicines and equipment  培训当地医务人员 Training of local healthcare professionals  技术合作与交流 Technical cooperation and exchange |
| 26 | 我国公共卫生对外援助与合作的进展和展望 Progress and Outlook of China’s Public Health Foreign Aid and Cooperation | 2022 | 黄璐璐，丁玮，陆申宁，施丹丹，官亚宜，王多全，吕山，李石柱 | 热带病与寄生虫学 Tropical Diseases and Parasitology | 否 No | 案例研究Case article | 多个非洲国家 Multiple African countries | 公共卫生体系建设 Public health  传染病防控 Control of infectious diseases  疟疾防控 Malaria control  血吸虫病防治 Schistosomiasis prevention  艾滋病防治 HIV/AIDS control  结核病防治 Tuberculosis control  卫生人力资源开发 Health workforce development | 公共卫生援助 Public health assistance  援建医疗卫生机构 Construction of medical facilities  提供药品和医疗设备 Supply of medicines and equipment  健康教育和健康促进 Health education and promotion  培训当地医务人员 Training of local healthcare professionals  免疫接种 Vaccination  技术合作与交流 Technical cooperation and exchange |
| 27 | 中国援非医疗队历史的再考察（1963—1983） Re-examining the History of China’s Medical Teams to Africa (1963–1983) | 2022 | 蒋华杰 | 外交评论 Foreign Affairs Review | 是 Yes | 案例研究Case article | 多个非洲国家 Multiple African countries | 卫生事业发展 Healthcare service enhancement  缺医少药 Lack of healthcare workers and medicines | 派遣医疗队 Medical team assistance |

**Table 5** Summary of key findings from reviewed Chinese articles

| **研究目标**  **Study objectives** | **归纳主题**  **Emerging theme(s)** | **主要发现**  **Main findings** |
| --- | --- | --- |
| 项目形态  Mortality | 项目地点  Programme location | - 医疗队通过改善社区诊所设施与机制强化基层卫生服务能力 Medical teams enhanced primary care by improving community clinics (Refs: 3, 4, 17, 21) - 实施下乡巡回医疗服务，确保农村地区居民获得基本医疗 Mobile services ensured rural access to basic care (Refs: 5, 14, 17) - 与当地对口医院合作开展定点支援，建设专科中心，提升区域医疗水平 Collaboration with local hospitals improved regional specialty care (Refs: 8, 9, 10) |
|  | 项目周期  Programme duration | - 部分医疗援助以“短期项目式医疗队”为主要形式，执行专项任务 Short-term project-based teams addressed targeted needs (Refs: 8, 14, 16) - 医疗项目兼顾短期任务与长期合作，包括“光明行”等短期援助与中外对口医院合作等长期形式 Combination of short- and long-term cooperation formats observed (Refs: 6, 13, 18, 24) |
|  | 合作机制  Collaborative initiatives | - 实施多层次援助，包括派遣医疗队、提供设备、建设专科医疗中心与药物供应 Multi-level support through team dispatch, equipment provision, and center building (Refs: 2, 6, 10, 14, 20) - 推动对口医院合作，建立专科中心，协同控制重大疾病 Specialized centers and joint disease control with partner hospitals (Refs: 8, 9, 13, 24) - 开展形式多样的中非卫生合作活动，包括交流培训、医院援建、义诊及药品捐赠等 Varied collaboration formats including training, construction, free clinics, donations (Refs: 9, 11, 12, 16, 23) |
|  | 资源与技术  Resources & expertise | - 举办专题讲座、同台手术及教学查房，为受援国医务人员提供技术培训与指导 Technical training through lectures, joint surgeries, ward rounds (Refs: 1, 5, 10, 13, 18, 22, 26) - 提供大型医疗设备与药品，提高受援国医疗资源配置水平 Provision of medical equipment and supplies (Refs: 2, 6, 9, 12, 20, 24) - 建议构建系统化、规范化的对外医疗援助机制以提升整体效率 Need for standardized and institutionalized aid mechanisms (Refs: 6, 11, 15, 19, 25) |
| 项目效果  Programme effectiveness | 满足当地医疗需求  Meeting local healthcare needs | - 提供常见病和多发病诊疗服务，满足基层医疗机构服务能力有限地区的基本诊疗需求 Provided basic diagnosis and treatment for common diseases in underserved areas (Refs: 1, 2, 8, 10, 13, 15, 19, 20) - 在应对突发公共卫生事件（如埃博拉和新冠疫情）中发挥了重要作用，缓解了当地医疗资源紧张的局面 Critical during public health emergencies (Refs: 6, 7, 18, 26) - 针对特定人群开展巡诊义诊、妇幼健康服务、慢病管理等，提升医疗服务可及性和公平性 Mobile clinics improved equity and access (Refs: 3, 5, 14, 16, 22) |
|  | 提升医疗服务质量  Improving the quality of care | - 医疗队引入中国先进诊疗理念和技术，提升当地诊疗水平和服务流程 Introduced advanced Chinese practices to improve diagnosis and workflows (Refs: 2, 4, 9, 17, 21) - 通过提供高质量外科手术、眼科治疗等专业服务，明显改善患者治疗结局和满意度 Specialized services (e.g., surgery, ophthalmology) improved outcomes (Refs: 8, 10, 19, 20, 23); - 强调中西医结合服务模式，有助于丰富当地医疗服务体系与技术手段 TCM-Western medicine integration expanded care options (Refs: 6, 11, 13, 24) |
|  | 能力建设  Capacity building | - 通过临床带教、技术指导、病例讨论等形式，提升当地医务人员专业技术水平 Improved clinical skills through bedside teaching, guidance, and case discussions (Refs: 1, 4, 9, 12, 15, 25) - 与当地医学院校合作开展师资培养、教材编写、继续教育，支持医学教育体系建设 Supported education through faculty training, textbook development, CME (Refs: 2, 5, 14, 17, 27) - 协助完善医院制度建设与管理流程，促进当地医疗机构规范化运行 Institutional development enhanced operational standards (Refs: 3, 7, 11, 16) |
| 实施促进因素  Major drivers | 政治与外交目标  Political and diplomatic goals | - 中国援非医疗队在国家外交战略中发挥作用，通过援外历史传承、制度安排和双边关系的推动，增强政治影响力与合作意愿 Strengthened political will through historical continuity and bilateral agreements (Refs: 1, 9, 11, 12, 19, 20, 23–26) - 中非医疗合作被纳入“一带一路”等国家战略中，体现全球卫生治理的政策导向 Aligned with China’s national strategies like the Belt and Road Initiative (Refs: 16, 24, 26) - 医疗援助被视为超越政治的外交工具，有助于增强国际认可与形象，拓展话语权 Seen as diplomatic soft power tools (Refs: 21–23, 26) |
|  | 经济合作  Economic cooperation | - 援非医疗行动促进医药产品与服务贸易、投资合作及中资企业海外布局 Promoted trade, investment, and overseas operations (Refs: 15, 23–25) - 民间经济联系与中非产业互动增强卫生援助的实用价值和互惠基础 Private sector ties added practical value (Refs: 9, 18, 23, 25) - 医疗保障提升被视为服务在非华人企业和华侨的重要手段，间接促进对外经济合作 Healthcare support aided Chinese expatriates (Refs: 23, 25) |
|  | 人道主义援助  Humanitarian aid | - 援外医疗队以改善非洲人民健康为出发点，体现中国的大国担当与人道主义精神 Demonstrated China’s commitment to global solidarity and well-being (Refs: 1, 14, 17, 21, 27) - 针灸等传统医学适用于资源匮乏地区，深受非洲人民欢迎，体现中医药的人道价值 TCM valued in low-resource settings (Refs: 1, 14, 21) |
| 实施阻碍因素  Barriers | 基础设施与资源差异  Infrastructure and resource disparities | - 受援国公共卫生条件差、医疗体制不完善以及援助项目管理与制度机制不健全，限制了项目实施效果 Weak health systems and poor public health conditions limited impact (Refs: 1–5, 7–15, 17–20, 22–27) - 持续的人力资源短缺问题限制了医疗援助的长期可持续性发展 HR shortages challenged sustainability (Refs: 17, 22) |
|  | 文化差异  Cultural differences | - 中非之间存在显著的文化、制度及医学观念差异，影响了中方医疗队的适应与融合 Differences in culture, systems, and medical concepts affected integration (Refs: 2, 4, 5, 7, 10, 11, 18, 26) |
|  | 语言障碍  Language barriers | - 语言沟通障碍在医疗服务交付中广泛存在，尤其在多语种地区对医疗效果造成一定影响 Communication challenges persisted, especially in multilingual regions (Refs: 4, 5, 7, 14, 26) |
